# Development of a first-contact protocol to guide assessment of adult patients in rehabilitation services networks

**DOI:** 10.1590/bjpt-rbf.2014.0137

**Published:** 2016-01-19

**Authors:** Mariana A. P. Souza, Fabiane R. Ferreira, Cibele C. César, Sheyla R. C. Furtado, Wendy J. Coster, Marisa C. Mancini, Rosana F. Sampaio

**Affiliations:** 1 Programa de Pós-graduação em Ciências da Reabilitação, Universidade Federal de Minas Gerais (UFMG), Belo Horizonte, MG, Brazil; 2 Departamento de Estatística, UFMG, Belo Horizonte, MG, Brazil; 3 Departamento de Fisioterapia, UFMG, Belo Horizonte, MG, Brazil; 4 Departament of Occupational Therapy, Boston University (BU), Boston, MA, United States; 5 Departamento de Terapia Ocupacional, UFMG, Belo Horizonte, MG, Brazil

**Keywords:** rehabilitation, assessment, patient-centered care, international classification of functioning, disability and health

## Abstract

**Objective:**

This paper describes the development of the Protocol for Identification of Problems for Rehabilitation (PLPR), a tool to standardize collection of functional information based on the International Classification of Functioning, Disability and Health (ICF).

**Development of the protocol:**

The PLPR was developed for use during the initial contact with adult patients within a public network of rehabilitation services. Steps to develop the protocol included: survey of the ICF codes most used by clinical professionals; compilation of data from functional instruments; development and pilot testing of a preliminary version in the service settings; discussion with professionals and development of the final version. The final version includes: user identification; social and health information; brief functional description (BFD); summary of the BFD; and PLPR results. Further testing of the final version will be conducted.

**Conclusions:**

The protocol standardizes the first contact between the user and the rehabilitation service. Systematic use of the protocol could also help to create a functional database that would allow comparisons between rehabilitation services and countries over time.

## BULLET POINTS

- Rehabilitation treatment should focus on the patient functional demands.
- The PLPR standardizes the data collected at the beginning of rehabilitation.
- Thus, it improves communication among professionals, services, and patients.
- It includes minimal sets of ICF codes, relevant for people with disabilities.
- The ICF codes will allow comparisons between services and locations over time.

## Introduction

Over the last centuries, the world has faced a global demographic transition, with an increase in life expectancy and in chronic health conditions, resulting in the emergence of new, increasingly complex, disability-causing illnesses, either transient or permanent^1^^,^ 2. These changes have been challenging health systems by increasing the demand for rehabilitation services^2^. The situation presents an ideal opportunity for the development of a consistent model of rehabilitative care that integrates these services across a continuum of care in the health system.

In Brazil, the strategy employed to deal with this new demand was to create multidisciplinary teams and to structure public rehabilitation services in an integrated network organized across three levels of care. Basic care is supported by Family Health Care Centers, whose services are delivered in the community, close to the family's residence and, in some cases, in the patient's home^3^^,^ 4. Specialized care is offered at Specialized Rehabilitation Centers, which are responsible for treatments that require higher technological support^3^. Finally, hospital care is responsible for handling persons with disabilities in urgent and emergency situations as well as assignment to in-patient rehabilitation beds^3^.

Given the diversity of services and professionals, these multidisciplinary teams need to have competencies beyond their specific professional skills. These competencies include good communication skills, the use of appropriate protocols and procedures that reflect the goals of the service, and an integrated focus on the needs of patients^5^. At the start of this project, the work of the rehabilitation networks was often marked by poor systematization in collecting and sharing information on the target population. Furthermore, the information gathered did not always reflect patients' functional condition, preventing construction of a database that would support proper administrative, organizational, and financial planning of rehabilitation services.

In order to overcome these inadequacies, beginning in 2012, the Brazilian Ministry of Health recommended the use of the International Classification of Functioning, Disability and Health (ICF) as a clinical and statistical tool in health services^6^. To implement the ICF in everyday services, professionals must adopt the biopsychosocial model as a guide for their actions and use evaluation and functioning data collection protocols that are consistent with the model^7^^-^ 9. Thus, a systematized approach to patients from the first contact with a rehabilitation service is important to enable correct identification of the patient's limitations in functioning.

In the context of public health in Brazil, the first contact of the individual with the health service represents a strategic moment as it guides the organization of health units and the work process. The first contact is the moment when the patient or family member first seeks the health service due to a specific health complaint - a health professional must listen to the patient's complaint and establish a therapeutic alliance. The goal is to guarantee admittance to everyone who seeks services from the public health system and to understand the needs of the individual so that each case is addressed in the most suitable way^10^. The first contact process is followed throughout the Unified Health System (*Sistema Único de Saúde - SUS*). At this first contact, collaboration is established between the patient and the health team, which brings the patient into the center of his or her own therapeutic process^11^^,^ 12. The therapeutic process can be understood as all of the treatments available to the patients through SUS (medical appointments, exams, medication, and others). The inclusion of the patient and his or her family in decisions concerning the therapeutic process has been associated with higher autonomy and accountability of the patient, increased compliance, and satisfaction with the treatment^13^.

Because the beginning of the rehabilitation treatment should focus on identifying the problems and needs of the individual, effective communication among all persons involved in the process is important to ensure a complete understanding of the patient's situation^8^. The first contact seems to be the right moment to use tools that help overcome professional boundaries and incorporate different perspectives that contribute to the improvement of shared decision-making in the rehabilitation treatment. The purpose of this paper is to describe the development process of an ICF-based protocol for collecting information during the initial contact with adult patients in rehabilitation services networks.

## Method

### Development of the protocol for identification of problems for rehabilitation

The Protocol for Identification of Problems for Rehabilitation (Protocolo de Levantamento de Problemas para a Reabilitação - PLPR) was developed through a partnership between researchers from Universidade Federal de Minas Gerais (UFMG), Belo Horizonte, MG, Brazil and professionals representing public rehabilitation services in Belo Horizonte in the year 2012. Belo Horizonte is one of the largest cities in Brazil^14^. The city has an extensive network of rehabilitation services across the three levels of care laid out in the legislation, which includes 58 community service centers, three centers of specialized care, and beds in 33 hospitals. These services involve multidisciplinary teams that include physical therapists, occupational therapists, speech pathologists, nutritionists, psychologists, social workers, pharmacists, and physical educators. In total, there are more than 500 professionals involved in these services^15^.

The PLPR was developed after a series of meetings that included 61 rehabilitation professionals and rehabilitation managers from the public rehabilitation services of Belo Horizonte, as well as rehabilitation researchers from UFMG. Each professional who participated in this group was selected by his or her immediate manager. The main goal of these meetings was to re-design the model of care of the public rehabilitation network of Belo Horizonte, in an attempt to follow the guidelines from the Ministry of Health^3^^,^ 6. The gatherings also provided an opportunity for this group to discuss and develop the protocol to facilitate the implementation of the proposed new model.

Development of the PLPR involved a series of steps: 1) survey of the ICF codes most frequently used by professionals in the public rehabilitation services; 2) compilation of information contained in functional instruments available in the literature; 3) development of a preliminary version of the protocol; 4) pilot testing of the preliminary version; 5) discussion with rehabilitation professionals; and 6) development of the final version of the protocol.

For the survey of ICF codes, the professionals were asked to assemble a list of the codes that were most frequently used in rehabilitation services in their workplace. To create this list, the professionals were instructed to confer with their colleagues (other rehabilitation professionals in the services) and to select the second-level ICF codes most often used in their clinical practice.

In addition, the following sources were also analyzed and reviewed in order to guide the selection of items to be included in the protocol: ICF Checklist; ICF Core Sets (e.g. Chronic Widespread Pain; Low Back Pain; and Stroke)^16^; functional evaluations already used by the professionals in the services (e.g. Functional Independence Measure - FIM; Visual Analogue Scale for Pain; and Medical Outcomes Study Short Form 36 - SF-36)^17^; and the *Minimal Generic Set* and the *Disability Set*, which are considered relevant to persons with disabilities^18^. The functional measures used as references to develop the protocol have already been linked to the ICF or were developed using the ICF model. To define which questions and codes should be included in the protocol, rehabilitation professionals, managers, and researchers were guided by daily practice in the services, including the most frequent patient requirements for rehabilitation, questions considered essential to decide which service and what kind of treatment the patient needs, and the protocol's feasibility (time to complete). After discussing these issues and analyzing all of the material, the codes that would comprise the PLPR were defined and guiding questions were written, resulting in a preliminary version of the instrument.

### Initial pilot testing

To check the feasibility of the protocol, a pilot test was carried out across the community service centers and centers of specialized care. The rehabilitation professionals who were participating in the development process were asked to apply the draft protocol to all patients seeking treatment at the rehabilitation services for one month. After that, the professionals were asked to meet with their team partners to discuss and record their experiences when using this version. Any concerns were then discussed among the researchers and professionals. According to the professionals, the time to complete the protocol varied from 15 to 30 minutes, decreasing as professionals became accustomed to using it. After this initial testing, revisions were made to the preliminary version of the protocol and a final version was developed. Further testing of the final version will be conducted and presented in subsequent studies.

## Results

### Final protocol for identification of problems for rehabilitation

The final version of the protocol consisted of four parts: 1) user identification; 2) social and health information; 3) brief functional description (BFD) and summary of the BFD; and 4) results. The user identification section included information such as name and health unit.

The social and health information section includes questions concerning risk factors and self-perception of emotional and physical health. This part also includes information about ICF environmental factors such as employment status, use of prosthetics and/or orthotics, the need for assistance from others to perform daily tasks, and ongoing health treatments.

The BFD was created based on sets of ICF codes considered relevant for people with a medical condition that causes disability or poses a risk for developing disability^18^. These sets of items are called the *Minimal Generic Set* (MGS) and the *Disability Set* (DS). The MGS corresponds to a set of seven codes proposed by the WHO to be used in surveys regarding disabilities and health. The DS is a set of 22 ICF codes, including the seven codes from the MGS and 15 more related to body function, activity, and participation. The DS codes are proposed as good descriptors of disability situations and are included in a project by the WHO and the World Bank^18^.

Based on the codes obtained from the rehabilitation professionals and on the professionals' experiences in the pilot test, the minimal sets proposed by WHO were expanded. Content considered important to the performance of different professionals in the multidisciplinary teams was added and codes thought to be less relevant to the reality of the services were removed. For example, we decided to remove the code "Sexual Functions" (b640) because it was found to be an uncomfortable question to ask the patients in the initial contact - most of them could not answer properly. On the other hand, we decided to add questions regarding communication skills (d330-Speaking; d350-Conversation) to identify whether the patient needed to be seen by a speech pathologist. Table 1 compares the codes in the sets suggested by the WHO and those in the preliminary and final versions of the BFD of the PLPR. The final version of the BFD comprises 25 codes distributed in 10 domains (Table 1).

**Table 1.** Comparison of the ICF codes in the Minimal Generic Set and Disability Set and in the versions of the Brief Functional Description.

**ICF codes in each domain**	**MGS and Disability Set (WHO)**	**BFD - Preliminary Version (PLPR)**	**BFD - Final Version (PLPR)**
Mobility			
b455 Exercise tolerance functions	✓	✓	✓
b710 Mobility of joint functions	✓	✓	✓
b730 Muscle power functions	✓	-	-
d410 Changing basic body position	-	-	✓
d450 Walking (G)	✓	✓	✓
d455 Moving around (G)	✓	✓	-
d470 Using transportation	✓	✓	✓
Communication			
d330 Speaking	-	✓	✓
d350 Conversation	-	✓	✓
Eutrophy			
b510 Ingestion functions	-	✓	✓
b530 Weight maintenance functions	-	✓	✓
Self-care			
d510 Washing oneself	✓	✓	✓
d530 Toileting	-	-	✓
d540 Dressing	✓	✓	✓
d570 Looking after one's health	✓	✓	✓
Pain			
b280 Sensation of pain (G)	✓	✓	✓
Interpersonal activity			
d710 Basic interpersonal interactions	✓	✓	✓
d920 Recreation and leisure	✓	✓	✓
Energy and Sleep			
b130 Energy and drive functions (G)	✓	✓	✓
b134 Sleep functions	✓	✓	✓
Affect			
b152 Emotional functions (G)	✓	✓	✓
b640 Sexual functions	✓	✓	-
d240 Handling stress and other psychological demands	✓	✓	✓
d770 Intimate relationships	✓	✓	✓
General tasks and demands			
d230 Carrying out daily routine (G)	✓	✓	✓
d640 Doing housework	✓	✓	✓ *
d660 Assisting others	✓	✓	✓ *
Remunerative employment			
d850 Remunerative employment (G)	✓	✓	✓ +

MGS: minimal generic set; DS: disability set; BFD: brief functional description. **(G) Codes in bold** represent the seven codes of the MGS. ✓ Code present. - Code absent. * After discussions with professionals, it was decided to transfer the codes d640 (Doing housework) and d660 (Assisting others) from the "Affect" domain to the "General tasks and demands" domain on the final version of the PLPR. + To better identify difficulties in performing tasks related to the remunerative work, it was decided to transfer the code d850 (Remunerative employment) from the "General tasks and demands" domain and create a "Remunerative employment" domain.

In order to standardize the use of the BFD codes, a guiding question was created for each of the 25 codes, based on the description in the ICF manual for each second-level code included in the BFD and their higher codes (see Appendix 1). For example, to create the guiding question for code b455 (Exercise tolerance functions), the ICF manual was consulted, and the descriptions of codes b455, b4550, b4551, and b4552 were analyzed, resulting in the question "*When engaging in physical effort, do you feel tired or short of breath?*".

To describe the individual's level of function and disability for each of the BFD codes, we used the ICF qualifiers. Thus, after asking the reference question for a particular BFD code, if the patient reported some difficulty in the situation represented by the code, he/she would be asked to quantify the difficulty on a 5-point scale from 0 (no disability or difficulty) to 4 (complete disability or difficulty). Therefore, it is the patient or his/her proxy who quantifies the extension of the problems in the questions of the BFD.

**Figure 1.**
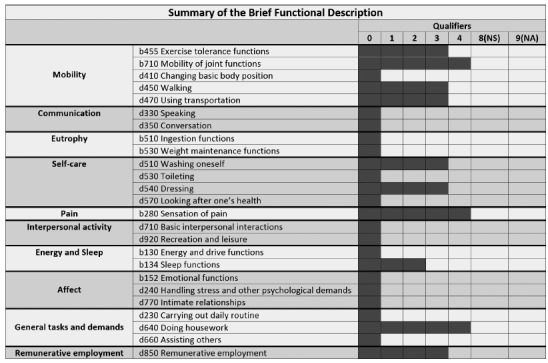
Example of summary of the brief functional description.

After initial pilot testing, the professionals reported that patients had difficulty comprehending the ordinal 0-4 rating scale. Consequently, a scale from 0 (no disability or difficulty) to 10 (complete disability or difficulty) was used instead, as patients were more familiar with this type of scale. Transformations of original ICF qualifiers to the 11-point scale were conducted using a conversion table present in the manual^19^. A visual analog scale was created for patients who reported difficulty understanding the BFD questions. This figure includes a graded color code and a numerical 0-10 scale grouped according to ICF qualifiers, with descriptive words for each qualifier. The professional chooses the format most suited to the patient's understanding in order to quantify the severity of his/her problem. After completion of the PLPR form, the professional fills out a table on the front page of the protocol (summary of the BFD), coloring in the spaces relative to the qualifier for each BFD question, which results in a graphic representation of the patient's main functional limitations (Figure 1).

A final BFD score summarizes the functional information reported by the patient. It varies from 0 to 100 points based on the sum of normalized sub-scores from each of the 10 BFD domains. Higher final scores represent lower functional level. Each BFD item is rated on a five-point scale according to the following ICF qualifiers: (0) no impairment or difficulty; (1) mild impairment or difficulty; (2) moderate impairment or difficulty; (3) severe impairment or difficulty; or (4) complete impairment or difficulty^19^. The two qualifiers (8) non-specified and (9) non-applicable receive a score of zero.

In order to normalize each BFD domain by their number of items and keep the same maximum score (10) across domains, a weight was created for each domain. For example, the mobility domain has 5 items and its raw score can vary from 0 to 20. By attributing a weight of 0.5 to this domain, its maximum raw score becomes 10. Furthermore, the pain domain, which has only one item that can be scored on a 0 to 4 scale, receives a weight of 2.5. The weights were created to normalize the impact of each domain on the protocol's final score. Table 2 shows the weights attributed to each BFD domain and gives an example of final score computation.

**Table 2.** Example of scoring in the Brief Functional Description.

**BFD Domains**	**Qualifier reported**	**Weight in the domain**	**Total score in the domain (sum of qualifiers in the domain × weight)**
Mobility			
b455 Exercise tolerance functions	3	0.5	13 × 0.5 = 6.5
b710 Mobility of joint functions	4		
d410 Changing basic body position	0		
d450 Walking	3		
d470 Using transportation	3		
Communication			
d330 Speaking	0	1.25	0 × 1.25 = 0
d350 Conversation	0		
Eutrophy			
b510 Ingestion functions	0	1.25	0 × 1.25 = 0
b530 Weight maintenance functions	0		
Self-care			
d510 Washing oneself	3	0.625	6 × 0.625 = 3.75
d530 Toileting	0		
d540 Dressing	3		
d570 Looking after one's health	0		
Pain			
b280 Sensation of pain	4	2.5	4 × 2.5 = 10
Interpersonal activity			
d710 Basic interpersonal interactions	0	1.25	0 × 1.25 = 0
d920 Recreation and leisure	0		
Energy and Sleep			
b130 Energy and drive functions	0	1.25	2 × 1.25 = 2.5
b134 Sleep functions	2		
Affect			
b152 Emotional functions	0	0.833	0 × 0.833 = 0
d240 Handling stress and other psychological demands	0		
d770 Intimate relationships	0		
General tasks and demands			
d230 Carrying out daily routine	0	0.833	4 × 0.833 = 3.33
d640 Doing housework	4		
d660 Assisting others	0		
Remunerative employment			
d850 Remunerative employment	3	2.5	3 × 2.5 = 7.5
TOTAL SCORE			33.58

The PLPR final score may help guide the planning of actions for the rehabilitation services network, contributing to the identification of intervention priorities for each patient and the necessary level of complexity.

The PLPR result is provided by the rehabilitation professional who completed the protocol (i.e. who received the patient in his/her first contact with the service). Based on the data collected with the PLPR, the professional identifies the patient's "primary need" for rehabilitation, the "professional indicated to coordinate the case" in the beginning of the rehabilitation process, and the "place to begin care" (i.e. in which service of the rehabilitation network the patient will start treatment). The rehabilitation location is determined primarily by the needs of the patients, availability of services, and professionals in a specific area of need, as well as issues such as the patient's ability to use public transportation safely. The coordinator of the case is responsible for optimizing patient flow across all points of the healthcare continuum, not only in rehabilitation. It is expected that rehabilitation professionals will be trained to apply the protocol and to use their clinical reasoning and experience to interpret the information gathered and make the best decision for each individual patient.

## Discussion

As in other countries, the public rehabilitation network in Brazil organizes its services across different levels of care, aiming to deliver integrated assistance to patients with diverse requirements^3^^,^ 20 ^-^ 22. In a truly integrated system, these services work together to organize efficient and effective patient flow. For this purpose, the services should work in an integrated manner with the existing health system. One of the key points of this model of care is that, although patients may need to access different services as they progress, their transition between sites should be optimized by communication and exchange of information between services so that patients can progress in an uninterrupted continuum of assistance across different levels of care^5^^,^ 20. A model of care for rehabilitation services should consider that this is not a linear process, and that the patients often need to visit and re-visit different points of the network as their recovery progresses and new challenges are faced. This requires integrated evaluations and a care coordinator to improve efficiency of the services and support achievement of positive patient outcomes^20^^,^ 23.

Another important issue is the difficulties encountered when introducing a new conceptual model to guide the actions of health services and adoption of these innovations in the daily routine of the services^24^. This is a challenge that requires considerable effort from professionals who usually must adapt to these changes without interrupting the care of patients under their responsibility. The PLPR was designed during meetings that aimed to re-design the model of care of a public rehabilitation network, and it is one of the strategies proposed for practical implementation of the transformations that result from adoption of the biopsychosocial ICF model^25^.

We expect that the PLPR can contribute to improving communication among professionals and services and guiding the patient's pathway throughout the rehabilitation network. The use of this protocol systematizes the information collected in the initial contact, ensuring that this information is available online to be accessed by professionals anywhere in the network. This standardization saves time and effort of professionals and patients. Based on the identified problems and needs, patients move more quickly to advanced stages of the rehabilitation process such as the use of specific evaluations after admission for treatment at the location indicated in the PLPR^8^^,^ 25. Thus, it is expected that more equitable access can be achieved in proper locations and in a timely fashion, contributing to greater effectiveness and efficiency of rehabilitation services.

Since the release of ICF, there has been considerable research focusing on its use in several contexts such as policies, statistics, and especially in the development of ICF-based assessment tools for clinical applications^26^^-^ 28. Apart from its specific application to intervention, availability of information about functioning is essential to policy planning, service planning, and investments in rehabilitation^29^^-^ 32. The PLPR aims to meet those needs by 1) identifying the functional needs of patients in a more systematic and informative way and 2) guiding the organization of services and the planning of rehabilitation actions.

The focus of the protocol on identifying the functioning concerns reported by the patient in the initial contact with the service is crucial. The use of this protocol from the start gives the patient and/or his/her family the opportunity to report his/her functional needs and expectations regarding the rehabilitation process. This promotes active participation in the patient's own treatment planning^13^. Hence, the PLPR has great potential to improve the organization of services by increasing patient compliance, as it considers the preferences of the patient and his/her functional needs to assist in the selection of the most appropriate professionals and services to initiate care from first contact.

In the development of the protocol, it is important to highlight the use of the minimal sets of ICF codes proposed by the WHO, as well as the participation of professionals from rehabilitation services. The inclusion of the minimal sets in the BFD will allow the comparison between the data collected with the PLPR and data from other services, locations, and at different times, as the WHO proposes the wide use of these minimal code sets in disability and health surveys^18^. Furthermore, by maintaining the majority of codes from those clusters in the protocol, it will be possible to merge databases based on the PLPR with other function-focused databases using specific statistical techniques (e.g. Item Response Theory)^33^.

The active participation of professionals in the construction of the PLPR led to a protocol that is in line with the reality of rehabilitation services and increased professional compliance when applied to the daily routines of services. However, innovations that require changes are not always easy, especially when they involve clinical practice, better collaboration among disciplines, or changes in the organization of care. Studies show that behavioral changes in clinical practice are possible, but require a comprehensive approach at different levels (hospitals, ambulatory, primary care) and adaptation to specific locations and groups, similar to what has been done in the development of the PLPR^24^.

In addition to the possibilities already described, the PLPR has also proved to be efficient in identifying patients who do not have a specific need for individualized care and who could take part in different group activities, undergo vocational guidance, and receive follow-up. Based on better identification of the functional needs of patients, as well as the best location to start the rehabilitation process, one might expect the use of the protocol to contribute to reducing the waiting list for rehabilitation care and the number of inappropriate transfers between services. These issues should be investigated in future studies.

## References

[1] Lee R (2003). The demographic transition: three centuries of fundamental
change. J Econ Perspect.

[2] World Health Organization - WHO (2011). World report on disability.

[3] Brasil. Ministério da Saúde (2012). Portaria GM/MS nº 793, de 24 de Abril de 2012. Institui a Rede de Cuidados à
Pessoa com Deficiência no âmbito do Sistema Único de Saúde.

[4] Brasil. Ministério da Saúde (2008). Portaria GM/MS nº 154, de 24 de Janeiro de 2008. Cria os Núcleos de Apoio à
Saúde da Família - NASF.

[5] Nancarrow SA, Booth A, Ariss S, Smith T, Enderby P, Roots A (2013). Ten principles of good interdisciplinary team work. Hum Resour Health.

[6] Brasil. Ministério da Saúde (2012). Resolução nº 452, de 10 de Maio de 2012. Estabelece que a Classificação
Internacional de Funcionalidade, Incapacidade e Saúde - CIF seja utilizada no
Sistema Único de Saúde, inclusive na Saúde Suplementar.

[7] Sampaio RF, Luz MT (2009). Funcionalidade e incapacidade humana: explorando o escopo da
classificação internacional da Organização Mundial da Saúde. Cad Saude Publica.

[8] Steiner WA, Ryser L, Huber E, Uebelhart D, Aeschlimann A, Stucki G (2002). Use of the ICF model as a clinical problem-solving tool in physical
therapy and rehabilitation medicine. Phys Ther.

[9] Rauch A, Cieza A, Stucki G (2008). How to apply the International Classification of Functioning,
Disability and Health (ICF) for rehabilitation management in clinical
practice. Eur J Phys Rehabil Med.

[10] Brasil. Ministério da Saúde (2006). Acolhimento nas práticas de produção de saúde.

[11] Franco TB, Bueno WS, Merhy EE (1999). O acolhimento e os processos de trabalho em saúde: o caso de Betim,
Minas Gerais, Brasil. Cad Saude Publica.

[12] Mitre SM, Andrade EIG, Cotta RMM (2012). Progress and challenges facing user acceptance in the implementation
and qualification of the Unified Health System in Primary Healthcare: a review of
the bibliographical output in Brazil. Cien Saude Colet.

[13] Rathert C, Wyrwich MD, Boren SA (2013). Patient-centered care and outcomes: a systematic review of the
literature. med Care Res Rev..

[14] Instituto Brasileiro de Geografia e Estatística - IBGE (2010). Censo Demográfico 2010.

[15] Belo Horizonte. (2013). Gerência de Reabilitação. Profissionais de Reabilitação nos Serviços da
Secretaria Municipal de Saúde de Belo Horizonte.

[16] ICF Research Branch (2013). ICF Core Sets Projects [Internet].

[17] Rehabilitation Institute of Chicago (2010). Rehabilitation Measures Database.

[18] Cieza A, Oberhauser C, Bickenbach J, Chatterji S, Stucki G (2014). Towards a minimal generic set of domains of functioning and
health. BMC Public Health.

[19] World Health Organization - WHO (2001). International Classification of Functioning, Disability and Health:
ICF.

[20] New SWH (2011). Rehabilitation Redesign Project Final Report [Internet].

[21] Holdsworth LK, Webster VS, McFadyen AK (2007). What are the costs to NHS Scotland of self-referral to physiotherapy?
Results of a national Trial. Physiotherapy.

[22] Nancarrow S, Moran A, Freeman J, Enderby P, Dixon S, Parker S (2009). Looking inside the black box of community rehabilitation and
intermediate care teams in the United Kingdom: an audit of service and
staffing. Qual Prim Care.

[23] Ireland H (2012). Rehabilitation following acquired brain injury: a headway review of
guidelines and evidence [Internet].

[24] Grol R, Grimshaw J (2003). From best evidence to best practice: effective implementation of
change in patient's care. Lancet.

[25] Sampaio RF, Ferreira FR, Souza MAP (2014). Reorientação do Modelo Assistencial da Rede de Reabilitação SUS.

[26] Cerniauskaite M, Quintas R, Boldt C, Raggi A, Cieza A, Bickenbach JE (2011). Systematic literature review on ICF from 2001 to 2009: its use,
implementation and operationalisation. Disabil Rehabil.

[27] Ruaro JA, Ruaro MB, Souza DE, Frez AR, Guerra RO (2012). An overview and profile of the ICF's use in Brazil: a decade of
history. Rev Bras Fisioter.

[28] Sampaio RF, Mancini MC, Gonçalves GGP, Bittencourt NFN, Miranda AD, Fonseca ST (2005). Aplicação da Classificação Internacional de Funcionalidade,
Incapacidade e Saúde (CIF) na prática clínica do fisioterapeuta. Rev Bras Fisioter.

[29] United Nations Economic and Social Commission for Asia and the Pacific -
UNESCO (2008). World Health Organization - WHO. Training Manual on Disability
Statistics.

[30] O'Donovan MA, Doyle A, Gallagher P (2009). Barriers, activities and participation: Incorporating ICF into service
planning datasets. Disabil Rehabil.

[31] Mueller M, Lohmann S, Strobl R, Boldt C, Grill E (2010). Patients' functioning as predictor of nursing workload in acute
hospital units providing rehabilitation care: a multi-centre cohort
study. BMC Health Serv Res.

[32] Disler PB, Roy CW, Smith BP (1993). Predicting hours of care needed. Arch Phys Med Rehabil.

[33] Jette AM, Haley SM (2005). Contemporary measurement techniques for rehabilitation outcomes
assessment. J Rehabil Med.

